# Task-Specific Multimodal Imaging and Spectroscopy for Post-Mortem Interval Assessment in Human Skeletal Remains: An Exploratory Decision-Support Framework

**DOI:** 10.3390/diagnostics16182908

**Published:** 2026-09-09

**Authors:** Johannes Dominikus Pallua, Bettina Zelger, Michael Schirmer, Anton K. Pallua, Galina Apostolova, Rohit Arora, Christian Wolfgang Huck, Claudia Wöss

**Affiliations:** 1 Department of Orthopaedics and Traumatology, Medical University of Innsbruck, 6020 Innsbruck, Austria; johannes.pallua@i-med.ac.at (J.D.P.); rohit.arora@i-med.ac.at (R.A.); 2 Institute of Pathology, Neuropathology, and Molecular Pathology, Medical University of Innsbruck, Muellerstrasse 44, 6020 Innsbruck, Austria; bettina.zelger@i-med.ac.at; 3 Institute of Legal Medicine, Medical University of Innsbruck, Muellerstraße 44, 6020 Innsbruck, Austria; 4 Institute of Analytical Chemistry and Radiochemistry, University of Innsbruck, 6020 Innsbruck, Austria; christian.w.huck@uibk.ac.at; 5 Department of Internal Medicine, Medical University of Innsbruck, 6020 Innsbruck, Austria; schirmer.michael@icloud.com; 6 Former Institute for Computed Tomography-Neuro CT, Medical University of Innsbruck, Anichstraße 35, 6020 Innsbruck, Austria; anton.k.pallua@cnh.at; 7 Institute for Neuroscience, Medical University of Innsbruck, 6020 Innsbruck, Austria; galina.apostolova@i-med.ac.at

**Keywords:** post-mortem interval, human skeletal remains, forensic medicine, micro-CT, Raman spectroscopy, near-infrared spectroscopy, hyperspectral imaging, multimodal diagnostics, hierarchical decision support, exploratory decision support, bone diagenesis

## Abstract

**Background/Objectives:** Estimating the post-mortem interval (PMI) of human skeletal remains remains challenging because bone undergoes structural, molecular and optical changes that evolve differently over time and are strongly influenced by taphonomic conditions. Rather than assuming that a single multimodal classifier performs equally well across all PMI intervals, this study aimed to determine which imaging or spectroscopic modality is most informative for specific forensic decision tasks and to derive an exploratory task-specific diagnostic decision-support framework based on internally evaluated sample-level analyses. **Methods:** Human femoral bone samples were assigned to five PMI classes ranging from 0–2 weeks to >100 years and examined using micro-computed tomography, hyperspectral imaging, handheld and microscopic Raman spectroscopy, and NIR-ONE spectroscopy. Repeated acquisitions were aggregated at the physical-sample level. Four targeted diagnostic contrasts were defined for the present reanalysis: archaeological class 5 versus classes 1–4, early classes 1 + 2 versus later classes 4 + 5, classes 1 + 2 versus class 4 within the Raman-compatible forensic range, and class 1 versus class 2. In addition, an exploratory direct pairwise comparison of class 4 versus class 5 was performed to specifically assess the boundary between the latest forensic interval and archaeological material. Parameter-wise ROC analysis, bootstrap confidence intervals, exploratory operating points at approximately 95% specificity, and repeated stratified cross-validation were used. All preprocessing for multivariate modelling was performed within the respective cross-validation folds. **Results:** Diagnostic performance was strongly task-dependent. Archaeological class 5 was distinguished from classes 1–4 by micro-CT Mean2 (AUC 0.984), NIR-ONE reflectance at 1944 nm (AUC 0.980), and HSI-derived tissue water index (TWI; AUC 0.961). In the additional exploratory direct C4-versus-C5 analysis, micro-CT Mean2 showed complete separation of the available samples (AUC 1.000), while NIR-ONE reflectance at 1944 nm retained excellent discriminatory performance (AUC 0.963). In contrast, HSI-derived TWI showed only moderate direct C4-versus-C5 discrimination (AUC 0.742). For early classes 1 + 2 versus later classes 4 + 5, the device-derived HSI StO_2_ index showed the highest univariate performance (AUC 0.940), followed by TWI (AUC 0.894) and the NIR spectral slope between 1550 and 1950 nm (AUC 0.860). Within the Raman-compatible forensic range, HSI remained highly informative, while Raman carbonate/phosphate and crystallinity parameters provided complementary molecular information. Class 1 versus class 2 discrimination remained moderate. Corrected five-class modelling achieved only moderate balanced accuracy and did not consistently improve upon NIR-ONE alone. **Conclusions:** Diagnostic performance was strongly task-dependent. In this internally evaluated cohort, micro-CT Mean2 and high-wavelength NIR-ONE features showed the strongest discrimination of archaeological-compatible C5 material, whereas HSI-derived optical indices were most informative for the separated early-versus-later contrast and Raman spectroscopy provided complementary molecular information within C1–C4. Because C5 comprised only six unique physical samples and archaeological context was confounded with chronological age, the very high C5-related AUC estimates should be regarded as exploratory, hypothesis-generating estimates rather than validated measures of chronological PMI. The proposed decision-support framework is likewise exploratory and requires independent external validation before forensic implementation.

## 1. Introduction

Estimating the post-mortem interval (PMI) of human skeletal remains is a central challenge in forensic medicine, forensic anthropology, and medico-legal death investigation. In skeletal remains, conventional PMI estimation is particularly difficult because soft-tissue-based indicators are no longer available and bone undergoes complex post-mortem changes influenced by biological, chemical, physical, and environmental factors. These processes include decomposition, diagenesis, mineral dissolution and recrystallisation, collagen degradation, hydration changes, microbial activity, soil composition, pH, temperature, humidity, burial conditions, and exposure to scavengers or weathering. Consequently, PMI estimation from skeletal remains has been imprecise, and no universally accepted single method is available for reliable dating across early forensic, later forensic, and archaeological time intervals [1,2,3].

Bone diagenesis is especially relevant for PMI assessment because post-mortem alteration affects both the organic and inorganic components of bone. The mineral phase, mainly represented by hydroxyapatite, and the organic matrix, especially collagen and associated proteins, may change at different rates depending on intrinsic bone properties and extrinsic environmental conditions. Histological studies have shown that post-mortem degradation can produce recognisable microscopic changes in bone tissue, but these alterations are highly context-dependent and may not be sufficient for precise PMI classification when used alone [4]. Experimental spectroscopic studies have likewise demonstrated that post-mortem diagenesis involves dynamic alterations of both the mineral and organic bone matrices and that these changes are influenced by environmental conditions [5]. Therefore, objective, reproducible, and preferably non-destructive methods are needed to complement conventional forensic assessment. As a consequence, in recent years imaging and spectroscopic techniques have gained increasing attention for PMI estimation because they provide different types of structural, chemical, molecular and spatial information from skeletal material.

Previous work has demonstrated that different analytical approaches capture distinct aspects of post-mortem bone alteration and may therefore provide complementary information rather than competing diagnostic outputs. Indeed, micro-computed tomography (micro-CT) provides high-resolution three-dimensional information on cortical bone architecture, porosity, bone volume fraction, and density-related parameters, and is therefore suited to assess structural degradation and mineral-density-related changes in human skeletal remains. Work from our group further indicated that parameters such as BV/TV and density-related grey-value measures may serve as candidate structural markers of post-mortem bone alteration. Thus micro-CT primarily reflects structural consequences of decomposition and diagenesis, and this approach may be limited when skeletal remains in early PMI categories show overlapping morphology [6].

Alternatively, vibrational spectroscopic techniques provide complementary molecular information. For example, Raman spectroscopy assesses mineral- and matrix-related bone components, including phosphate, carbonate, amide, and collagen-associated bands. Previous Raman-based studies demonstrated that PMI-associated spectral changes can be detected in bone tissue and that chemometric analysis may support discrimination between PMI categories [7]. Our previous work comparing handheld and microscopic Raman spectroscopy indicated that even portable Raman systems detect relevant mineral-associated PMI signatures, particularly in the phosphate region around 953–960 cm^−1^ [7]. At the same time, these studies highlighted important limitations, including fluorescence interference, spectral variability, and overlap between adjacent PMI classes. Raman spectroscopy therefore provides biologically interpretable molecular information, but may require a combination with additional analytical methods for robust multiclass PMI estimation [8,9].

Another technique, near-infrared (NIR) spectroscopy, represents a rapid, non-destructive point-based optical approach as NIR spectra contain broad overtone and combination bands related to O–H, C–H, and N–H bonds. Thus NIR spectroscopy may reflect PMI-associated changes in hydration, organic matrix composition, and mineral-associated properties. Previous work with handheld NIR spectrometry showed promising results for the classification of human skeletal remains into PMI categories using machine-learning approaches, supporting the feasibility of rapid optical PMI screening. Nevertheless, NIR spectroscopy alone may be limited in resolving closely adjacent early PMI intervals because early PMI classes may share overlapping hydration- and matrix-related spectral characteristics [10]. However, the previous NIR study primarily addressed overall class prediction, whereas the present reanalysis evaluates wavelength-specific and task-specific diagnostic behaviour at the physical-sample level.

Finally, hyperspectral imaging (HSI) extends point-based spectroscopy by combining spectral and spatial information. This enables surface-level visualisation of optical heterogeneity and the generation of device-derived spectral parameter maps conventionally labelled as perfusion, oxygenation/StO_2_, tissue haemoglobin, and water indices. In post-mortem skeletal material, these outputs should be interpreted as system-derived optical indices rather than physiological measurements. Previous HSI work demonstrated that classification performance increased with longer PMI and was particularly strong for differentiating archaeological material from forensic samples. Thus, HSI may be especially valuable as a rapid first-line spatial screening modality, whereas detailed molecular or structural confirmation may still be required in ambiguous cases [11]. The present study therefore re-analysed HSI-derived sample-level parameters, including oxygenation/StO_2_, tissue haemoglobin index and tissue water index, rather than relying solely on previously reported image-classification outputs.

Taken together, previous modality-specific studies indicate that micro-CT, Raman spectroscopy, NIR spectroscopy and HSI capture distinct aspects of post-mortem bone alteration. However, it remains unclear whether their diagnostic value is uniform across the full PMI range or depends on the specific forensic question. The present retrospective multimodal study therefore aimed to perform a harmonised physical-sample-level reanalysis and to address four task-specific questions: (i) which parameters are most informative for distinguishing archaeological class 5 from classes 1–4; (ii) which modalities best differentiate early PMI classes 1 + 2 from later classes 4 + 5; (iii) which Raman-derived molecular parameters are informative for differentiation within the forensic range, particularly classes 1 + 2 versus class 4; and (iv) whether class 1 can be reliably distinguished from class 2. Given the forensic relevance of distinguishing later forensic from substantially older skeletal material, an additional exploratory pairwise analysis directly comparing class 4 and class 5 was also performed to assess this specific temporal boundary. We hypothesised that diagnostic performance would be task-dependent and that a selective hierarchical workflow would be more informative than a uniform all-modality classifier.

## 2. Materials and Methods

### 2.1. Study Design

This study was designed as a retrospective, multimodal diagnostic study to develop and internally evaluate a hierarchical framework for PMI estimation in human skeletal remains. The study integrated structural, molecular, point-spectroscopic, and spatial hyperspectral information obtained from micro-CT, Raman spectroscopy, near-infrared spectroscopy using the NIR-ONE platform, and hyperspectral imaging (HSI). Microscopic Senterra Raman spectroscopy was considered as an exploratory complementary Raman modality within the overall five-method framework. The primary analyses were organised around forensically interpretable binary contrasts rather than a single global multiclass endpoint.

The four principal task-specific contrasts were specified before calculation of the corresponding task-specific ROC and model-performance results in the present harmonised reanalysis and were based on the predefined PMI class structure and forensic interpretability rather than on optimisation of observed discrimination. C1 and C2 jointly represent the first six months after death, whereas C4 and C5 represent clearly later forensic and archaeological-compatible intervals in the present classification scheme. C3 (>6 months–1 year) lies directly between these windows and was therefore retained in descriptive class-wise analyses but was not assigned to either side of the deliberately separated early-versus-later contrast. This design was intended to evaluate a high-level triage question and does not imply that C3 represents a biologically homogeneous or diagnostically irrelevant interval. Because omission of the intermediate class can increase apparent separation between the remaining groups, performance from this contrast should not be extrapolated directly to an unselected population spanning the complete PMI continuum. The additional direct C4-versus-C5 comparison was performed after the primary task-specific analyses and is therefore explicitly considered post-analysis, exploratory, and hypothesis-generating.

Four targeted contrasts were defined for the present harmonised reanalysis: class 5 versus classes 1–4, classes 1 + 2 versus classes 4 + 5, classes 1 + 2 versus class 4 for Raman-compatible analyses, and class 1 versus class 2. Class 3 was retained in descriptive class-wise analyses but treated as a transition interval and excluded from the targeted early-versus-later binary contrast. In addition, an exploratory direct pairwise comparison of class 4 versus class 5 was performed after the primary task-specific analyses to specifically examine the boundary between the latest forensic interval and archaeological material. This analysis was considered exploratory because it was motivated by the forensic interpretation of the pooled C5-versus-C1–C4 results and because the number of class-5 samples was small.

### 2.2. Human Bone Samples and PMI Classification

The analysed material consisted of human femoral diaphyseal cortical bone samples with assigned PMI categories, originating from forensic and archaeological contexts and available across the underlying modality-specific datasets [6,7,10,11,12]. The samples originated from forensic and archaeological contexts and were categorised into the following five PMI classes: (1) 0–2 weeks, (2) >2 weeks–6 months, (3) >6 months–1 year, (4) >1–10 years, and (5) >100 years. The PMI classification was based on available forensic case information, conventional assessment, and previously established class definitions. Samples were prepared as transverse bone sections, and the periosteum and bone marrow were removed where required for modality-specific measurements. Before spectroscopic and imaging analyses, samples were air-dried at room temperature. The master sample mapping identified 107 unique physical samples with valid PMI-class assignments: class 1, *n* = 33; class 2, *n* = 47; class 3, *n* = 11; class 4, *n* = 10; and class 5, *n* = 6. Modality availability differed because not every physical sample had been examined using every technique. Therefore, all modality-specific and multimodal analyses report their respective analysis-ready sample sizes. Repeated measurements, spectra and regions of interest were aggregated at the physical-sample level before statistical modelling.

The harmonised cohort consisted of femoral diaphyseal cortical bone samples prepared as transverse sections. Because the present study retrospectively integrates modality-specific legacy datasets, donor age, sex, and detailed skeletal disease or pathology were not consistently available for every unique physical sample and were therefore neither imputed nor inferred. For HSI, measurements were acquired under standardised conditions at a fixed camera-to-sample distance of 50 cm; however, a harmonised numerical observation-angle variable was not available across the retrospective dataset. Consequently, anatomical sampling level and available acquisition geometry are reported, whereas complete donor-level demographic and pathology characteristics cannot be summarised reliably for all 107 samples.

### 2.3. Ethical Considerations

The underlying sample collection and analyses were conducted in accordance with the Declaration of Helsinki and applicable institutional requirements. Ethical approval for the analysis of human skeletal remains was obtained from the local ethics committee of the Medical University of Innsbruck under the approval number EK 1357/2021. The archaeological samples were included in accordance with the permissions and institutional regulations applicable to the respective collections.

### 2.4. Analytical Imaging and Spectroscopic Methods

The analytical methods applied in this study were grouped into structural imaging, molecular spectroscopy, point-based near-infrared (NIR) spectroscopy, and spatial hyperspectral imaging. Detailed device specifications, acquisition settings, calibration procedures and modality-specific measurement protocols for micro-CT, Raman spectroscopy, NIR-ONE spectroscopy and hyperspectral imaging have been reported in the corresponding original publications [6,7,10,11], whereas the harmonised physical-sample-level preprocessing and task-specific statistical reanalysis were performed specifically for the present study.

#### 2.4.1. Micro-CT

Micro-CT was used to assess quantitative parameters for structural and density-related properties of cortical bone. The predefined micro-CT parameter set included Mean1, Mean2, bone volume fraction (BV/TV), cortical porosity, trabecular number and trabecular separation. These variables represented density-related and architectural properties of cortical and trabecular bone and were analysed consistently across the available samples. Additional voxel- and porosity-related variables were reviewed as exploratory structural markers.

#### 2.4.2. Raman Spectroscopy

Raman spectroscopy was used to characterise post-mortem alterations of the mineral and organic bone matrices. Handheld Mira Raman spectra and microscopic Senterra Raman spectra were analysed separately. Raw spectra were preprocessed using endpoint baseline correction, Savitzky–Golay smoothing with a 15-point window and area normalisation. Repeated spectra were aggregated at the physical-sample level.

Quantitative Raman analysis was restricted to PMI classes 1–4 because spectra from class 5 showed excessive fluorescence that obscured the Raman signal and prevented reliable peak extraction. The analysis-ready class distribution was reported separately for each Raman platform.

For repeated cross-validated Raman modelling, the primary feature set was defined before model fitting and contained six biologically interpretable parameters: phosphate-associated intensity I958, crystallinity index 1/FWHM958, mineral-to-matrix ratio A958/A1656, carbonate-to-phosphate ratio A1070/A958, mineral-carbonate-related ratio A1070/A1450, and Amide-I-associated intensity I1656. No LASSO, recursive feature elimination, or other automated data-driven variable-selection algorithm was applied to this primary six-parameter model. Additional interpretable Raman variables, including Amide-III-associated intensity, were evaluated in parameter-wise exploratory analyses. Senterra-derived A577/A958 was retained as an explicitly post-hoc exploratory parameter and was not treated as a prespecified primary model variable.

Class-wise Raman spectra were used for descriptive visualisation, while targeted ROC analysis and repeated stratified cross-validation were restricted to contrasts for which valid Raman data were available. In particular, Raman was assessed for classes 1 + 2 versus class 4 and for class 1 versus class 2. Raman was not included in any quantitative task involving class 5.

#### 2.4.3. Near-Infrared Spectroscopy Using the NIR-ONE Platform

NIR-ONE spectra covered the wavelength range from 1550 to 1950 nm at 2-nm intervals. A total of 36,164 individual acquisitions were available. Repeated acquisitions were averaged at the physical-sample level before statistical analysis, resulting in 104 unique NIR-ONE samples, of which 100 could be mapped unambiguously to the master PMI metadata. Four unmapped samples were excluded.

Both raw sample-level spectra and standard normal variate-transformed spectra were evaluated. Wavelength-specific analyses focused on reflectance at 1944 nm, reflectance at 1586 nm and the spectral slope R1950–R1550. For multivariate modelling, the complete 201-variable sample-level NIR-ONE spectrum was retained as the input spectral vector, without supervised wavelength selection. Scaling and, where applicable, SNV preprocessing were followed by principal component analysis retaining a fixed 10 principal components. PCA was fitted exclusively using the training data within each cross-validation split, and the corresponding held-out samples were projected into the PCA space defined by that training fold.

#### 2.4.4. Hyperspectral Imaging

Hyperspectral imaging was performed using the previously established acquisition protocol [11]. The HSI dataset comprised co-registered maps of NIR perfusion, oxygenation/StO_2_, RGB appearance, tissue haemoglobin index (THI), and tissue water index (TWI). Because the measurements were performed on post-mortem, air-dried bone, these HSI-derived output parameters were interpreted as device-derived spectral indices rather than as physiological measurements. Accordingly, the labels NIR perfusion, oxygenation/StO_2_, THI, and TWI refer to the established nomenclature of the HSI system and do not imply actual tissue perfusion, physiological oxygen saturation, haemoglobin concentration, or physiological water status in the investigated bone samples. Samples were measured three times at a fixed camera-to-sample distance of 50 cm under standardised acquisition conditions. The original HSI acquisition documentation comprised 125 acquisition records. After reconciliation with the central sample metadata and aggregation of repeated acquisition records at the physical-sample level, 107 unique physical samples were available for the present HSI analysis. The class-specific distribution was C1, *n* = 33; C2, *n* = 47; C3, *n* = 11; C4, *n* = 10; and C5, *n* = 6. Thus, the HSI sample-level dataset corresponded to the complete master cohort, whereas the number of analysis-ready samples varied in individual targeted comparisons according to the PMI classes included. For the present sample-level reanalysis, three standardised regions of interest were defined on representative cortical bone surfaces using the RGB image and were transferred consistently to the corresponding HSI parameter maps. Regions containing background, glare, shadow, cut-edge artefacts, visible contamination, or damaged areas were excluded. For each region of interest, the corresponding device-derived NIR perfusion, oxygenation/StO_2_, THI, and TWI index values were recorded. ROI-derived measurements were aggregated at the physical-sample level by calculating the mean, standard deviation, minimum, maximum, and range for each HSI parameter. In addition, two exploratory ratios derived from the device-generated perfusion and THI indices were evaluated. The direct Perfusion/THI ratio was calculated only for samples with THI > 0, whereas the bounded index Perfusion/(Perfusion + THI) was calculated for all samples to avoid instability caused by zero or near-zero THI values. The resulting sample-level HSI parameters were used for descriptive class-wise analyses, parameter-wise ROC analyses, single-modality modelling, and matched multimodal analyses. The present sample-level reanalysis was based on standardised HSI parameter maps generated from the original hyperspectral image data rather than on the proprietary raw hyperspectral image cubes.

### 2.5. Statistical Analysis

Continuous variables are reported as medians and interquartile ranges. Class-wise differences were assessed using Kruskal–Wallis tests. Pairwise non-parametric comparisons were performed using Mann–Whitney U tests, and false-discovery-rate adjustment was applied within each modality where multiple related comparisons were performed. Spearman rank correlations were used to evaluate ordered class trends. All statistical tests were two-sided.

Targeted discrimination was quantified using receiver operating characteristic (ROC) analysis and the area under the ROC curve (AUC). Ninety-five per cent confidence intervals for selected ROC AUC estimates were obtained using 20,000 stratified bootstrap resamples at the physical-sample level, with a fixed random seed of 20,260,811 to ensure reproducibility. Exploratory operating points were determined using a constrained high-specificity criterion rather than Youden’s J statistic. Candidate thresholds were restricted to those achieving a specificity of at least approximately 0.95, after which the threshold yielding the highest sensitivity within this constrained set was selected. Because achievable specificity values are discrete in small datasets, the realised specificity could exceed 0.95. These operating points were generated solely to illustrate the internal sensitivity–specificity trade-off and were not interpreted as externally validated forensic cut-offs.

Single-modality and multimodal multivariate models were evaluated using repeated stratified cross-validation. Five folds were used where permitted by the minority-class size, with 20 repetitions. The independent statistical unit was the unique physical bone sample; repeated measurements, spectra, regions of interest, and technical replicates were aggregated at the physical-sample level before modelling. All fold-dependent preprocessing steps, including scaling, standard normal variate transformation where applicable, and principal component analysis, were fitted exclusively using the corresponding training fold and subsequently applied unchanged to the held-out fold. This procedure was used to prevent information leakage between training and evaluation data.

The primary low-dimensional model inputs were predefined, biologically interpretable feature sets rather than variables selected through an automated supervised feature-selection procedure. The micro-CT model used six predefined structural and density-related parameters, the HSI model used four primary device-derived sample-level optical indices, and the primary Mira and Senterra Raman models each used six predefined molecular parameters. No LASSO, recursive feature elimination, or comparable automated outcome-driven variable-selection algorithm was applied to these primary feature sets. Additional individual parameters were investigated separately in exploratory univariate analyses and were not retrospectively substituted into the predefined primary models on the basis of their observed performance.

NIR-ONE spectroscopy was handled differently because each sample-level spectrum contained 201 reflectance variables covering 1550–1950 nm in 2-nm increments. For multivariate NIR-ONE modelling, the complete spectral vector was retained without supervised wavelength selection. Dimensionality reduction was performed using principal component analysis with a fixed specification of 10 retained principal components. Scaling, standard normal variate preprocessing where applicable, and PCA were fitted exclusively to the training data within each cross-validation split, and held-out samples were projected into the PCA space defined by the corresponding training fold. Raw and SNV-preprocessed NIR-ONE models were evaluated separately.

Linear support-vector machines with class-balanced weights were used to provide a consistent supervised modelling framework across modalities. The SVM regularisation parameter was fixed at C = 1.0, corresponding to the implementation default, and was not optimised using a data-driven hyperparameter search. Consequently, no inner hyperparameter-selection procedure or nested cross-validation was performed for C. Model settings were not selected on the basis of performance in the held-out evaluation folds. All fold-dependent preprocessing steps, including scaling, SNV transformation where applicable, and PCA, were fitted exclusively on the corresponding training fold and subsequently applied unchanged to the held-out fold.

The available cohort size was determined by retrospective physical-sample availability rather than by a prospective power calculation. Conventional events-per-variable heuristics developed primarily for regression modelling are not directly transferable to PCA-reduced support-vector classifiers; nevertheless, predictor dimensionality was deliberately restricted relative to the available number of independent samples. The primary HSI model contained four predictors, the primary micro-CT and Raman models contained six predictors each, and the 201-variable NIR-ONE spectrum was reduced to 10 principal components before classification. Despite these dimensionality controls, the relatively small and imbalanced cohort, particularly the six C5 samples, entails a substantial risk of model instability and overfitting. All multivariate classification results were therefore regarded as exploratory internal estimates requiring independent external validation.

Single-modality models were evaluated separately for micro-CT, HSI, NIR-ONE, Mira Raman spectroscopy, and Senterra Raman microscopy. Raman spectroscopy was excluded from C5-containing quantitative models because C5 spectra were not quantitatively evaluable owing to excessive fluorescence. Multimodal models were restricted to physical samples with complete feature availability for every modality included in the respective model. For the corrected global five-class HSI + micro-CT + NIR-ONE analysis, complete information was available for 97 of the 107 unique physical samples; consequently, 10 samples (9.3%) were excluded from this complete-case model because at least one required modality block was unavailable. For the targeted C1 + C2-versus-C4 + C5 analysis, 96 physical samples were eligible after exclusion of C3, and 87 of these had complete HSI, micro-CT, and NIR-ONE information; thus, 9 of 96 task-eligible samples (9.4%) were excluded owing to incomplete modality availability.

No imputation of entirely missing modality blocks was performed. Missingness predominantly reflected non-acquisition or technical non-evaluability of an analytical modality rather than isolated missing scalar values within an otherwise complete measurement. Imputation of an entire imaging or spectroscopic modality would therefore require strong assumptions regarding relationships between analytically distinct data sources and could introduce synthetic cross-modal information. Complete-case multimodal estimates were consequently interpreted specifically for the corresponding analysis-ready subsets, and potential selection bias resulting from differential modality availability was considered when comparing model performance.

Because the PMI classes were imbalanced, balanced accuracy was defined as the primary multivariate performance metric. Accuracy and macro-F1 score were reported as secondary metrics for all applicable models, whereas ROC AUC, sensitivity, and specificity were additionally reported for binary classification tasks. For parameter-wise analyses, Mann–Whitney U statistics, ROC AUC values, bootstrap confidence intervals where applicable, and exploratory high-specificity operating points were used to characterise task-specific discrimination. The corrected global five-class analysis was treated as a secondary benchmark rather than as the primary diagnostic endpoint because the principal objective of the study was to evaluate task-specific analytical strengths across distinct forensic questions.

For each targeted comparison, the analysis-ready sample size depended on the PMI classes and modalities included in the respective task. For HSI, *n* = 107 samples were available for C5 versus C1–C4, *n* = 96 for C1 + C2 versus C4 + C5, *n* = 90 for C1 + C2 versus C4, and *n* = 80 for C1 versus C2. Modality-specific sample sizes differed according to analytical availability and are therefore reported together with the corresponding results rather than assuming identical cohorts across modalities.

All reported performance estimates represent internal evaluation only. Parameter-wise ROC analyses were performed at the physical-sample level with bootstrap confidence intervals where applicable, whereas multivariate classifiers were assessed using repeated stratified cross-validation. No independent external validation cohort was available. Consequently, the reported AUC values, operating points, and multivariate classification metrics should be interpreted as exploratory estimates of discrimination within the present cohort and not as externally validated measures of forensic diagnostic performance.

### 2.6. Development of the Exploratory Decision-Support Framework

The hypothesis-generating exploratory decision-support framework was derived after completion of the targeted analyses and was not prespecified or evaluated as a validated sequential classifier. For each forensic task, the modality or parameter with the strongest internally evaluated performance was identified. Modalities were then ordered according to diagnostic performance, non-destructiveness, acquisition burden, biological interpretability and sample availability. The resulting framework distinguished between rapid screening, archaeological C5 assessment, early-versus-later PMI assessment, molecular characterisation within classes 1–4 and probabilistic early-PMI refinement.

## 3. Results

### 3.1. Dataset Structure, Method Availability, and Multimodal Overlap

**Figure 1:**
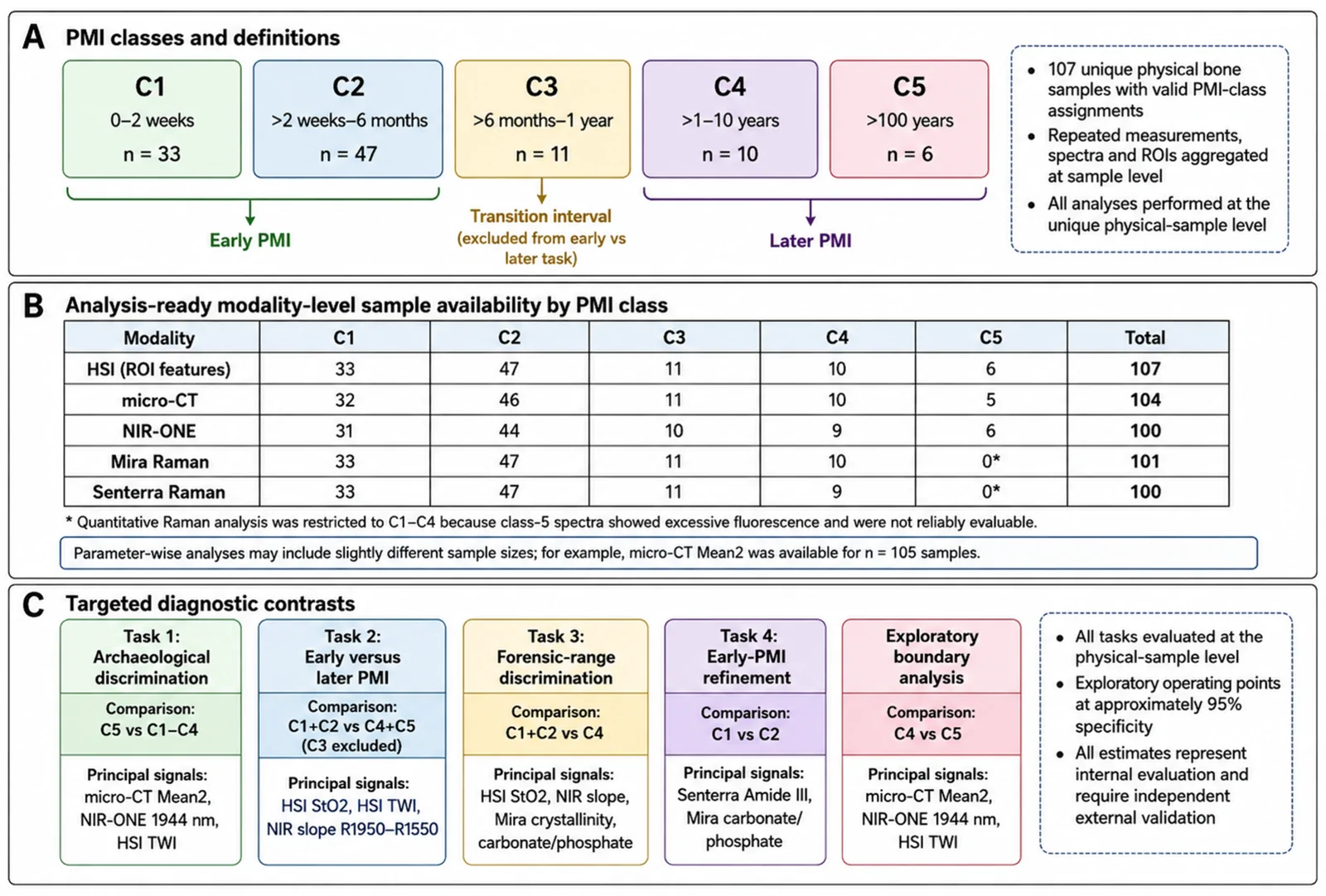
Study design, analysis-ready modality availability, and targeted diagnostic contrasts. (**A**) Definition of the five PMI classes: C1, 0–2 weeks; C2, >2 weeks–6 months; C3, >6 months–1 year; C4, >1–10 years; and C5, >100 years. C3 was treated as a transition interval and excluded from the targeted early-versus-later comparison. (**B**) Analysis-ready modality-level sample availability by PMI class for HSI-derived ROI features, micro-CT, NIR-ONE, Mira Raman, and Senterra Raman. Quantitative Raman analysis was restricted to C1–C4 because class-5 spectra showed excessive fluorescence. Parameter-specific sample sizes could differ slightly from modality-level counts; micro-CT Mean2, for example, was available for *n* = 105 samples. (**C**) Targeted diagnostic contrasts: C5 versus C1–C4, C1 + C2 versus C4 + C5, C1 + C2 versus C4, C1 versus C2, and the exploratory C4-versus-C5 boundary analysis. All analyses were performed at the physical-sample level and require independent external validation. Blue text indicates the principal internally evaluated analytical signal(s) for the respective diagnostic task.

The master sample mapping comprised 107 unique physical samples with valid PMI-class assignments: class 1, *n* = 33; class 2, *n* = 47; class 3, *n* = 11; class 4, *n* = 10; and class 5, *n* = 6. Modality availability differed because the underlying datasets originated from modality-specific acquisition campaigns. Repeated measurements, spectra, regions of interest and technical replicates were aggregated at the physical-sample level before analysis. Figure 1 summarises the PMI class definitions, modality-specific sample availability and the targeted diagnostic contrasts.

Modality-specific analyses used all valid samples available for the respective technique. For HSI, all 107 unique physical samples of the master cohort were available after aggregation of repeated acquisition records. Task-specific HSI sample sizes differed only because individual contrasts excluded classes not relevant to the respective forensic question. Multimodal analyses were restricted to the intersection of samples with complete data for the included modalities. Therefore, the analysis-ready sample size varied across tasks and models and is reported separately for each analysis.

For the corrected global five-class HSI + micro-CT + NIR-ONE model, complete modality information was available for 97 of the 107 unique physical samples; 10 samples (9.3%) were therefore excluded from this complete-case model because at least one required modality block was unavailable. For the targeted C1 + C2-versus-C4 + C5 analysis, 96 samples were eligible after exclusion of C3, and 87 of these had complete HSI, micro-CT, and NIR-ONE information; thus, 9 of 96 task-eligible samples (9.4%) were excluded owing to missing modality availability. Missingness predominantly represented non-acquisition or technical non-evaluability of an entire modality rather than isolated missing scalar values.

### 3.2. Exploratory Discrimination of Archaeological-Compatible Class 5 Material from Classes 1–4

Class 5 showed marked differences in structural, near-infrared and HSI-derived parameters. Micro-CT Mean2 achieved an AUC of 0.984, NIR-ONE reflectance at 1944 nm achieved an AUC of 0.980, and HSI TWI achieved an AUC of 0.961 for distinguishing class 5 from classes 1–4. Importantly, class 4 represented the latest forensic interval in the present classification scheme (1–10 years) and was included in the C1–C4 reference group. Thus, the strong C5-versus-C1–C4 discrimination indicates that the characteristic C5 pattern remained distinguishable despite inclusion of later forensic C4 samples in the comparator group, supporting analytical differentiation between later forensic and archaeological-compatible skeletal material. However, because the primary contrast pooled classes 1–4, these results should not be interpreted as performance estimates from a dedicated pairwise C4-versus-C5 classifier. More generally, the high AUC values observed in analyses involving C5 should be interpreted as exploratory estimates of discrimination within the present cohort rather than as validated chronological PMI performance. The small number of C5 samples results in substantial statistical uncertainty, and the observed separation may additionally reflect archaeological context and associated taphonomic characteristics. Accordingly, the C5-related findings are best regarded as hypothesis-generating evidence of an archaeological-compatible analytical pattern.

At the exploratory operating point requiring approximately 95% specificity, micro-CT Mean2 reached a sensitivity of 1.000 and specificity of 0.980, while NIR-ONE reflectance at 1944 nm reached a sensitivity of 0.833 and specificity of 0.989. HSI TWI achieved a sensitivity of 0.833 and specificity of 0.960 at the corresponding exploratory high-specificity operating point. These results indicate strong internal discrimination of archaeological class-5 material from the combined forensic reference group. Nevertheless, confidence intervals and thresholds remain unstable because class 5 contained only six unique physical samples, and the reported operating points should therefore not be interpreted as externally validated forensic cut-offs.

**Figure 2:**
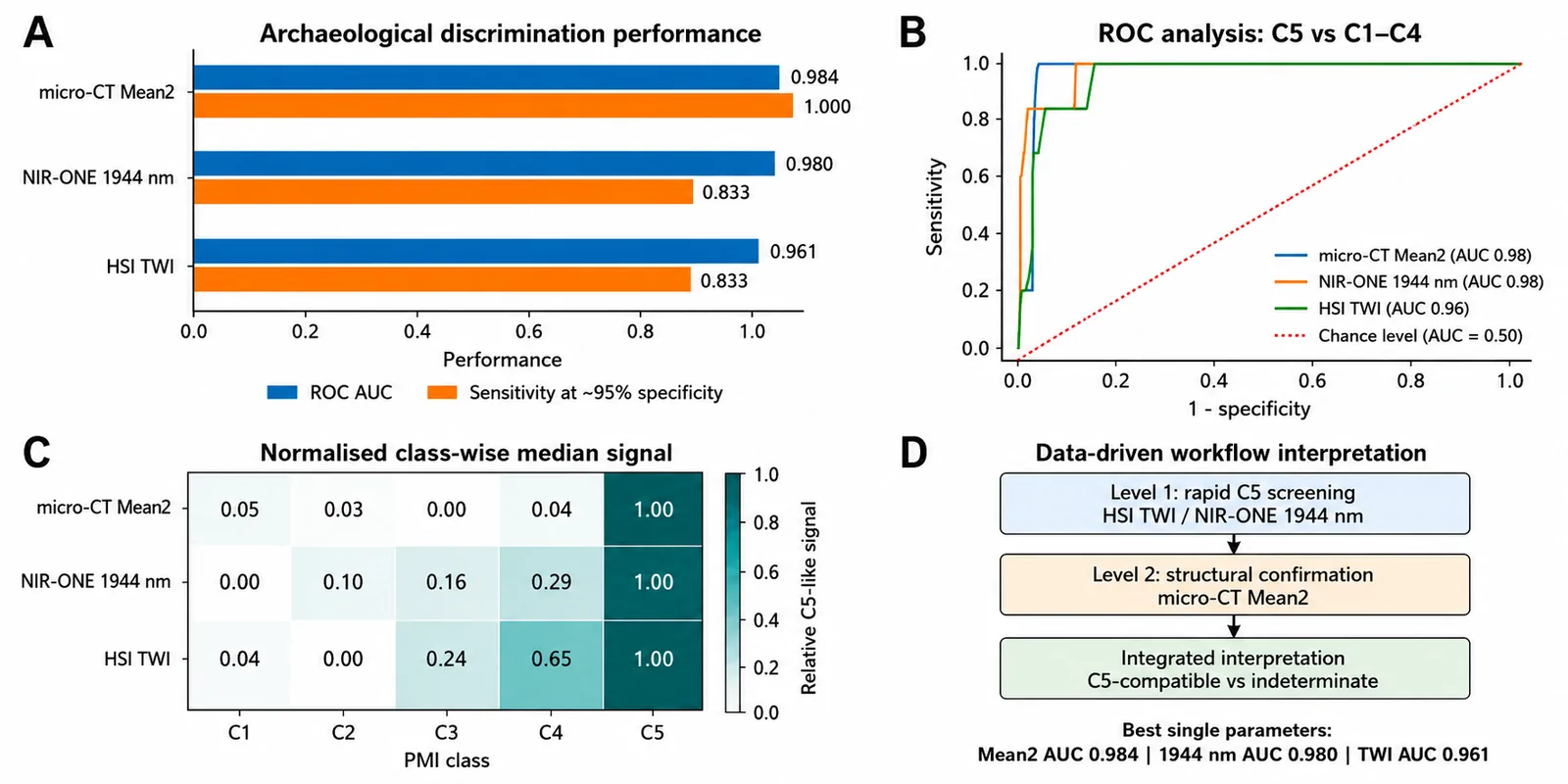
Exploratory discrimination of archaeological-compatible class-5 material. (**A**) Comparison of the diagnostic performance of micro-CT Mean2, NIR-ONE reflectance at 1944 nm, and the hyperspectral imaging-derived tissue water index (HSI TWI) for distinguishing class 5 (>100 years) from classes 1–4. Bars show the receiver operating characteristic area under the curve (ROC AUC) and the sensitivity obtained at an exploratory operating point requiring approximately 95% specificity. (**B**) ROC curves for class 5 versus classes 1–4. The dashed diagonal line indicates chance-level discrimination. The red dotted diagonal line indicates chance-level discrimination (AUC = 0.50). (**C**) Normalised class-wise median signals for the three best-performing parameters across PMI classes C1–C5, oriented so that higher values represent a stronger class-5-like pattern. (**D**) Data-driven interpretation of the proposed diagnostic workflow, in which rapid screening with HSI TWI and/or NIR-ONE at 1944 nm is followed, where required, by structural confirmation using micro-CT Mean2. Because C5 comprised only six unique physical samples, with only five C5 samples available for micro-CT Mean2, and because archaeological context cannot be separated from chronological age in the present cohort, all C5-related performance estimates and operating points should be regarded as exploratory, hypothesis-generating findings requiring independent external validation before forensic application.

To specifically examine the boundary between the latest forensic interval and archaeological material, an additional exploratory pairwise analysis of C4 versus C5 was performed. In the available samples, micro-CT Mean2 showed complete observed separation between C4 and C5, corresponding to an AUC of 1.000 (C4, *n* = 10; C5, *n* = 5). This finding should not be interpreted as evidence of perfect diagnostic performance, because complete separation is particularly susceptible to instability in such a small sample. In contrast, HSI-derived TWI showed only moderate direct C4-versus-C5 discrimination (AUC 0.742; 95% bootstrap CI, 0.475–0.983; C4, *n* = 10; C5, *n* = 6). Thus, the strong pooled C5-versus-C1–C4 performance of HSI TWI did not translate into comparably strong discrimination of the specific C4–C5 boundary. Given the small number of C5 samples, these pairwise findings remain exploratory and require independent validation. The task-specific discrimination results for archaeological-compatible C5 material and the corresponding exploratory workflow are summarized in Figure 2.

### 3.3. Early-Versus-Later PMI Discrimination

For the targeted comparison of classes 1 + 2 versus classes 4 + 5, HSI-derived StO_2_ achieved the highest univariate performance with an AUC of 0.940. This comparison represents a deliberately separated-window analysis rather than classification across the complete PMI continuum. C3 was retained for descriptive class-wise analyses but was neither reassigned nor included in this binary contrast. Consequently, the resulting AUC estimates quantify discrimination between non-adjacent PMI windows and may overestimate the performance expected in an unselected case mix containing intermediate C3 samples or continuously distributed PMI values. HSI TWI achieved an AUC of 0.894, and the NIR-ONE spectral slope R1950–R1550 achieved an AUC of 0.860. At the exploratory high-specificity operating point, the device-derived HSI StO_2_ index achieved a sensitivity of 0.625 and a specificity of 0.975, whereas HSI-derived TWI achieved a sensitivity of 0.813 and a specificity of 0.950. The derived Perfusion/(Perfusion + THI) index demonstrated a class-dependent trend but was retained as an exploratory parameter.

### 3.4. HSI-Derived Optical Indices and Perfusion/THI Relationships

**Figure 3:**
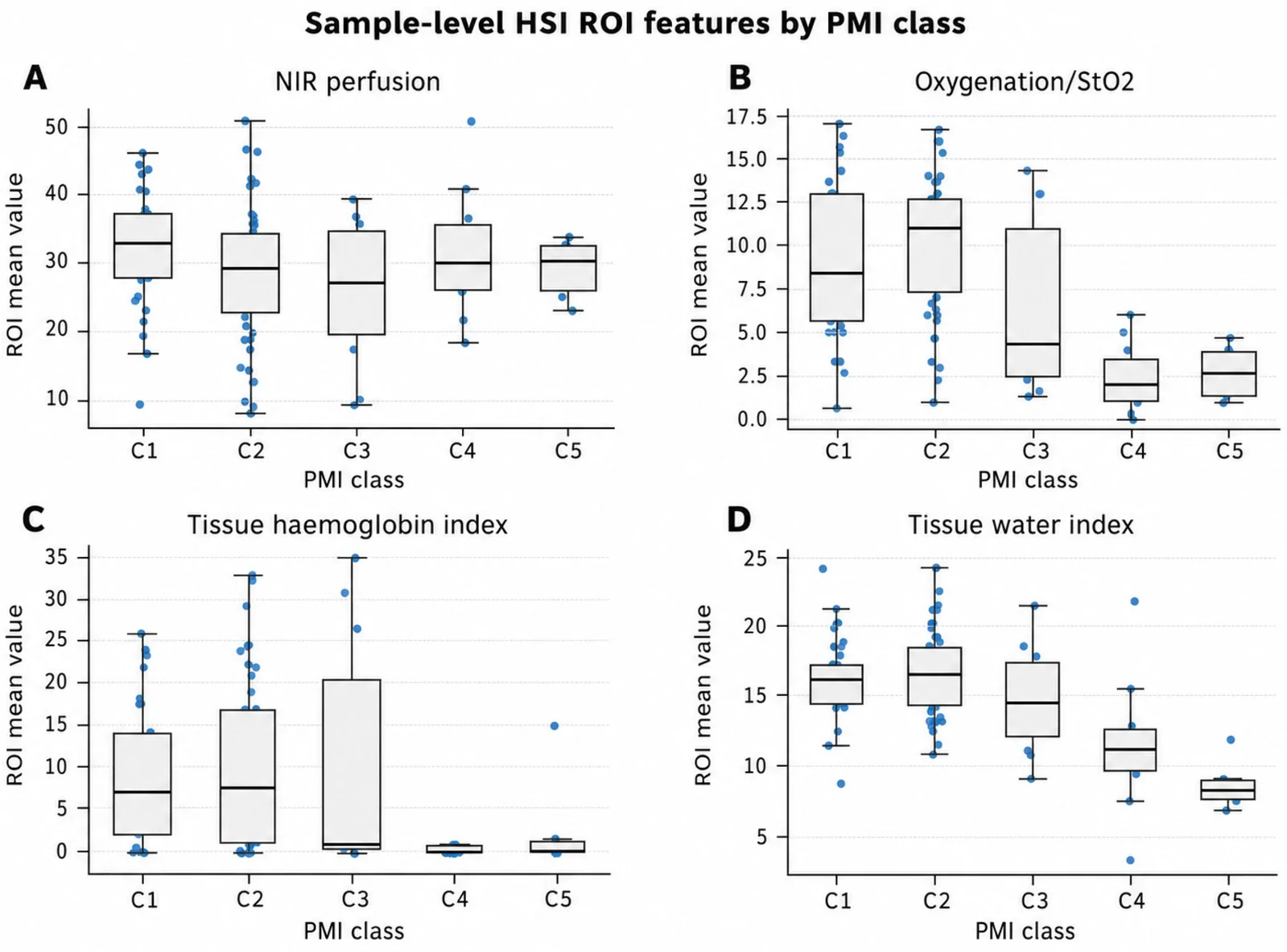
Sample-level HSI ROI features across PMI classes. For each bone sample, three standardised ROIs were evaluated and aggregated at the sample level. Boxplots show class-wise distributions of ROI-derived mean values for (**A**) NIR perfusion, (**B**) oxygenation/StO_2_, (**C**) tissue haemoglobin index, and (**D**) tissue water index. The HSI-derived parameters were evaluated as interpretable sample-level optical features in targeted task-specific analyses. All HSI parameters represent device-derived optical indices and should not be interpreted as physiological perfusion, oxygen saturation, haemoglobin concentration, or tissue water content in post-mortem bone.

HSI-derived ROI features were evaluated to assess whether spatial surface-level optical information contributes relevant information to multimodal PMI estimation. For each bone sample, three standardised ROIs were defined on representative cortical surface regions and transferred to the corresponding HSI parameter maps. ROI-derived values for NIR perfusion, oxygenation/StO_2_, tissue haemoglobin index, and tissue water index were aggregated at the physical sample level. These class-wise patterns (Figure 3) represent differences in device-derived optical indices and should not be interpreted as physiological perfusion, oxygen saturation, haemoglobin concentration, or tissue water content. To address the hypothesised relationship between perfusion and haemoglobin-associated indices, the direct Perfusion/THI ratio was examined in samples with THI > 0. Because zero THI values occurred frequently, particularly in later PMI classes, a bounded Perfusion/(Perfusion + THI) index was additionally calculated. The derived index showed a directional increase in later classes but was less diagnostically informative than StO_2_ and TWI. Therefore, StO_2_ and TWI were retained as the primary HSI parameters. These findings indicate that HSI-derived parameters should not be interpreted as isolated definitive PMI markers. However, StO_2_ and TWI showed direct task-specific value for differentiating early from later PMI intervals. Their principal contribution in the present dataset was therefore not merely as components of a fused model, but as interpretable optical parameters within a defined forensic contrast.

### 3.5. Raman-Derived Molecular Parameters Across Classes 1–4

**Figure 4:**
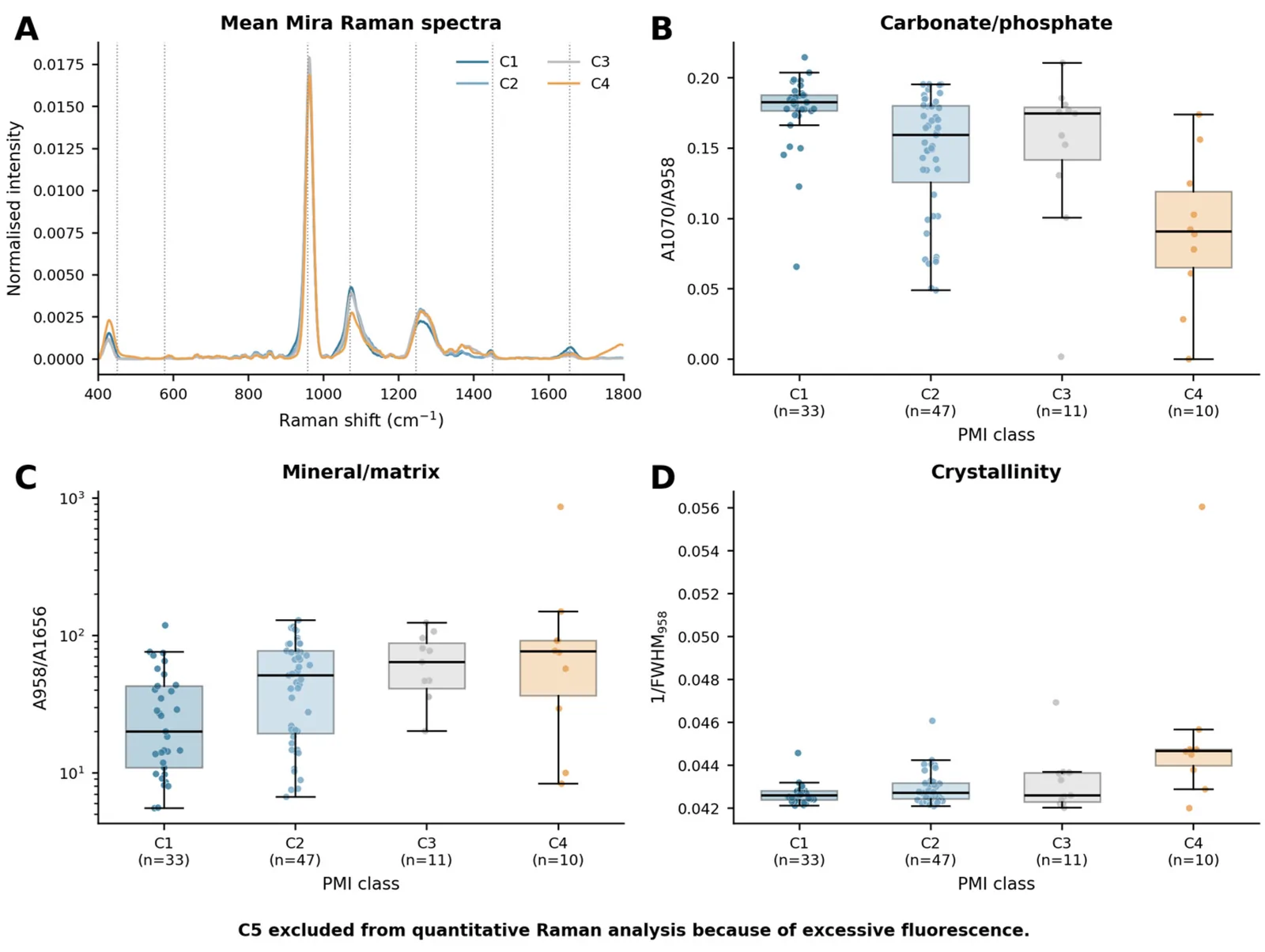
Raman-derived molecular characteristics across post-mortem interval classes 1–4. (**A**) Mean Mira Raman spectra for classes 1–4, with principal phosphate-, carbonate-, amide- and CH_2_-associated spectral regions indicated. (**B**) Carbonate-to-phosphate ratio A1070/A958, reflecting relative carbonate substitution within the mineral phase. (**C**) Mineral-to-matrix ratio A958/A1656, shown on a logarithmic scale because of the skewed parameter distribution. (**D**) Crystallinity index calculated as 1/FWHM958. Boxplots show the median and interquartile range, and individual points represent unique physical bone samples. Quantitative Raman analysis was restricted to classes 1–4 because excessive fluorescence in class-5 spectra prevented reliable peak extraction. The Raman-derived parameters provided complementary molecular information for forensic-range differentiation, particularly classes 1 + 2 versus class 4, whereas discrimination between class 1 and class 2 remained moderate.

Quantitative Raman analysis was restricted to classes 1–4 because excessive fluorescence prevented reliable parameter extraction in class 5. Class-wise mean spectra showed changes in phosphate-, carbonate- and amide-associated regions. For classes 1 + 2 versus class 4, Mira crystallinity achieved an AUC of 0.854, the Mira carbonate/phosphate ratio achieved an AUC of 0.849, and the Senterra carbonate/phosphate ratio achieved an AUC of 0.849. The Senterra A577/A958 ratio showed high exploratory performance but was considered post hoc and was therefore not used as a primary diagnostic parameter. For class 1 versus class 2, Raman performance was moderate, with Senterra Amide III reaching an AUC of 0.787 and Mira carbonate/phosphate reaching an AUC of 0.738. Class-wise mean Raman spectra and the distributions of the principal mineral- and matrix-associated Raman parameters are shown in Figure 4.

### 3.6. NIR-ONE Spectral Signatures

**Figure 5:**
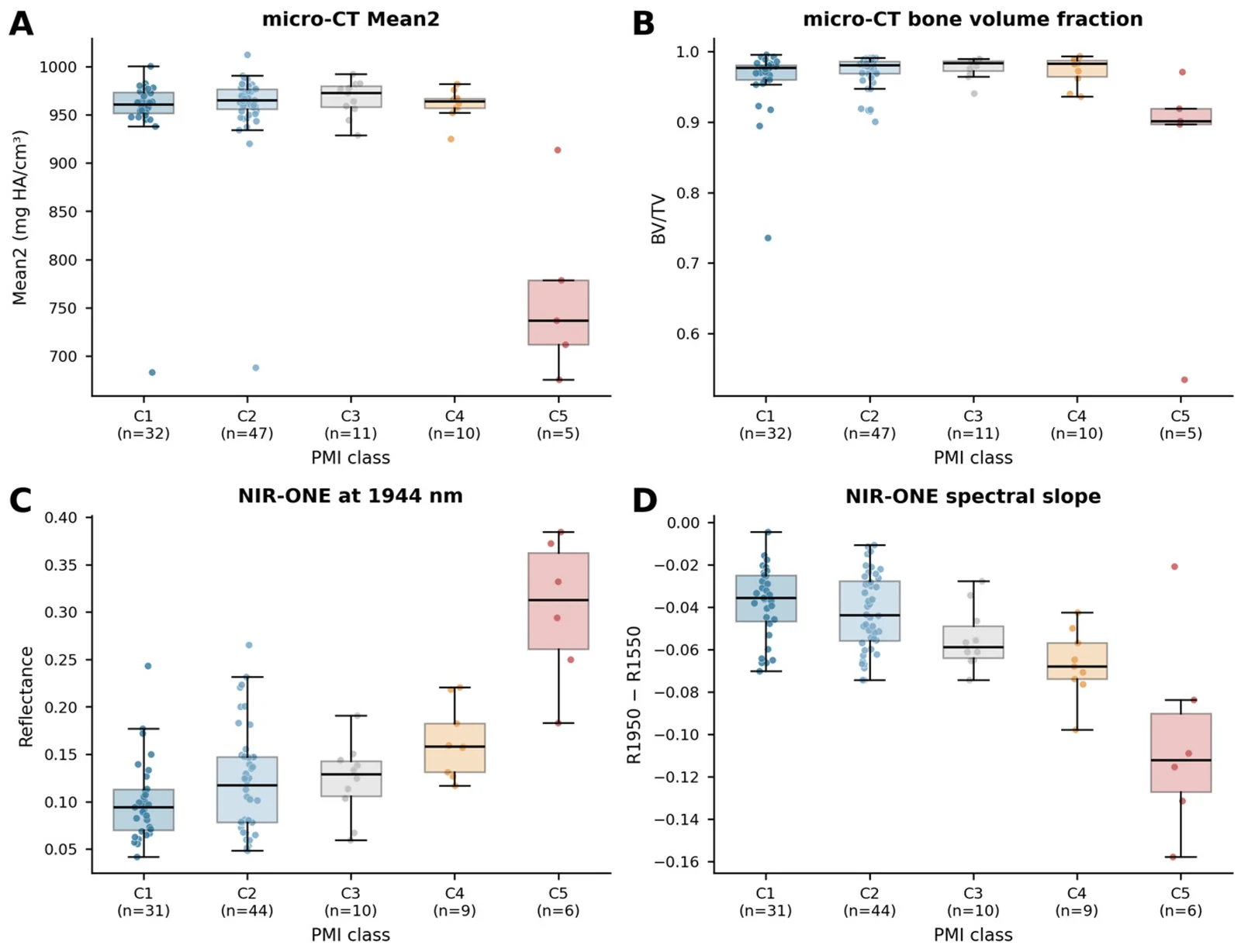
Class-wise distributions of selected micro-CT and NIR-ONE parameters across post-mortem interval classes. (**A**) micro-CT Mean2, representing a density-related structural parameter; (**B**) micro-CT bone volume fraction (BV/TV); (**C**) NIR-ONE reflectance at 1944 nm; and (**D**) the NIR-ONE spectral slope calculated as R1950–R1550. Boxplots show the median and interquartile range, whiskers represent the data range according to the boxplot definition, and individual points represent unique physical bone samples. Micro-CT Mean2 and NIR-ONE reflectance at 1944 nm showed the strongest differentiation of archaeological class 5 from classes 1–4, whereas the NIR spectral slope provided complementary information for differentiating early classes 1 + 2 from later classes 4 + 5. All parameter-specific findings represent internal analyses and require independent external validation.

NIR-ONE spectra demonstrated task-specific wavelength behaviour. Reflectance at 1944 nm showed the strongest discrimination of class 5 from classes 1–4, with an AUC of 0.980. For classes 1 + 2 versus classes 4 + 5, the spectral slope R1950–R1550 achieved an AUC of 0.860. In contrast, NIR-ONE discrimination of class 1 versus class 2 remained moderate. Wavelength-resolved AUC analysis showed that the 1944-nm finding represented a broader high-wavelength discriminatory region rather than an isolated single-point effect. The class-wise distributions of the most informative micro-CT and NIR-ONE parameters are shown in Figure 5.

### 3.7. Task-Specific Cross-Method Comparison

**Figure 6:**
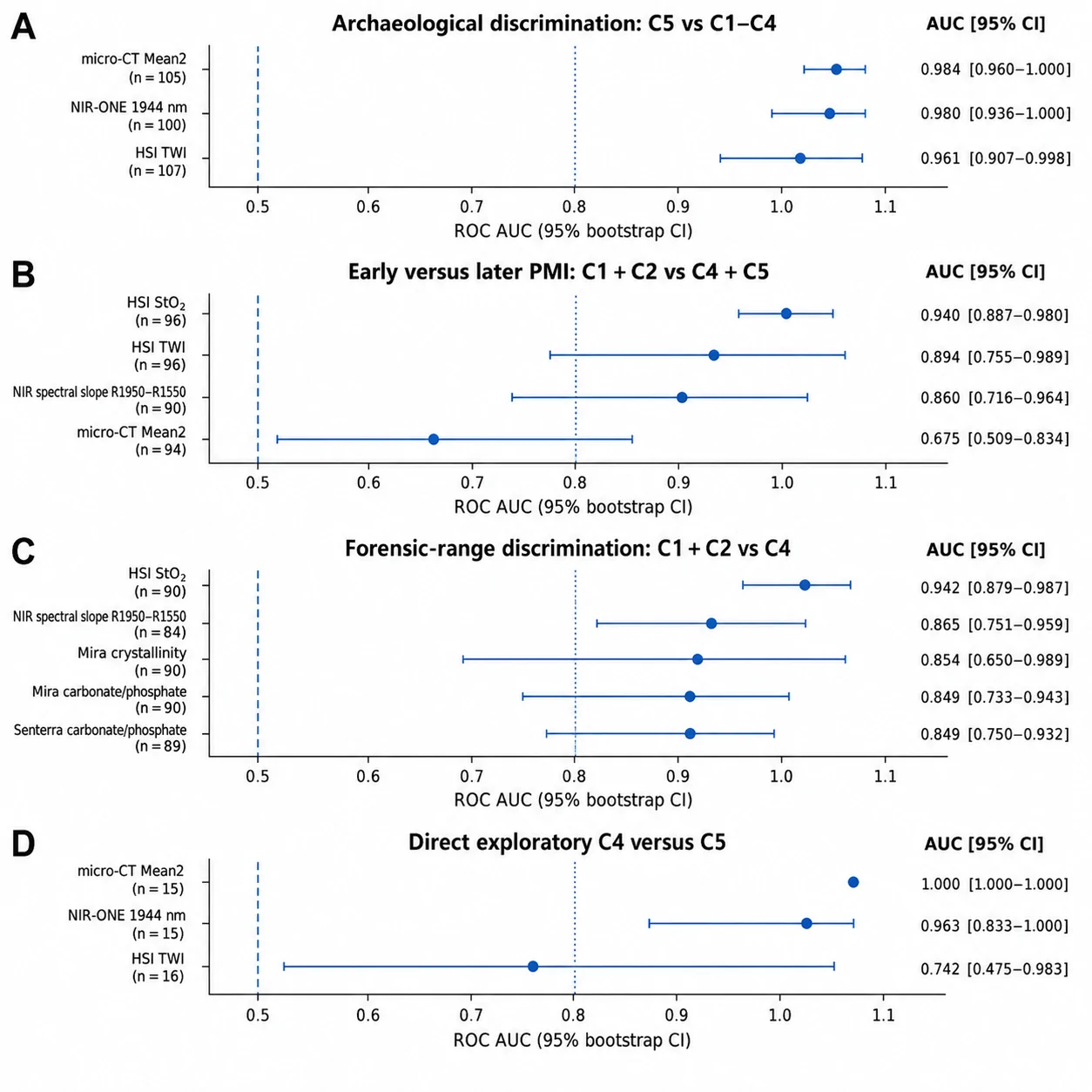
Task-specific diagnostic performance across imaging and spectroscopic modalities. Forest plots show ROC AUC estimates with 95% bootstrap confidence intervals for four diagnostic comparisons. (**A**) Exploratory discrimination of archaeological-compatible C5 material from the C1–C4 reference group using micro-CT Mean2, NIR-ONE reflectance at 1944 nm, and HSI-derived tissue water index (TWI). (**B**) Early-versus-later PMI discrimination (C1 + C2 vs. C4 + C5) using the device-derived HSI StO_2_ index, HSI TWI, the NIR-ONE spectral slope R1950–R1550, and micro-CT Mean2; C3 was excluded as a transition interval. Accordingly, Panel B represents a deliberately separated-window contrast and should not be interpreted as performance across the complete continuous PMI range. (**C**) Forensic-range discrimination (C1 + C2 vs. C4) using the HSI StO_2_ index, NIR-ONE spectral slope R1950–R1550, Mira Raman crystallinity and carbonate/phosphate ratio, and Senterra Raman carbonate/phosphate ratio. (**D**) Exploratory direct C4-versus-C5 discrimination using micro-CT Mean2, NIR-ONE reflectance at 1944 nm, and HSI TWI. Points indicate AUC estimates and horizontal lines the corresponding 95% bootstrap confidence intervals; analysis-ready sample sizes are shown for each parameter. The dashed line denotes chance-level discrimination (AUC = 0.50), and the dotted line marks AUC = 0.80 as a visual reference. Panel (**D**) is exploratory because of the small C5 sample size and post-analysis parameter selection. All estimates require independent external validation before forensic implementation.

Cross-method comparison confirmed that no single modality was uniformly optimal across all forensic tasks. For the distinction between archaeological class 5 and classes 1–4, micro-CT Mean2 achieved the highest AUC of 0.984, followed by NIR-ONE reflectance at 1944 nm with an AUC of 0.980 and HSI TWI with an AUC of 0.961. For early classes 1 + 2 versus later classes 4 + 5, the device-derived HSI StO_2_ index showed the strongest univariate performance with an AUC of 0.940, followed by HSI TWI with an AUC of 0.894 and the NIR-ONE spectral slope R1950–R1550 with an AUC of 0.860. Within the Raman-compatible forensic range, HSI StO_2_ remained highly informative for classes 1 + 2 versus class 4, while Mira Raman crystallinity and carbonate/phosphate parameters provided complementary molecular evidence. These task-specific performance patterns are summarised in Figure 6. The modality-specific estimates above were obtained from the maximum analysis-ready sample set available for each technique and therefore should not be interpreted as a strict head-to-head comparison performed on one identical set of physical samples. A universal matched comparison including micro-CT, HSI, NIR-ONE, and Raman was not possible for C5-inclusive tasks because quantitative Raman parameters could not be obtained from C5 owing to excessive fluorescence. Within the Raman-compatible C1 + C2-versus-C4 comparison, repeated cross-validated single-modality models showed balanced accuracies of 0.828 for the four-parameter HSI model (*n* = 90), 0.764 for the predefined six-parameter Mira Raman model (*n* = 90), and 0.726 for the NIR-ONE raw PCA10 model (*n* = 84), with corresponding mean ROC AUC values of approximately 0.945, 0.867, and 0.801, respectively. Thus, HSI remained numerically strongest in this forensic-range task, but the exact rank order should still be regarded as availability-dependent rather than as an invariant hierarchy across analytical technologies.

Overall, structural and high-wavelength near-infrared parameters were most informative for archaeological discrimination, optical HSI parameters were strongest for early-versus-later differentiation, and Raman parameters contributed primarily to molecular refinement within classes 1–4.

### 3.8. Secondary Global Five-Class Modelling

Corrected global five-class models achieved only moderate performance. NIR-ONE SNV with PCA achieved the highest single-modality balanced accuracy of approximately 0.483, while the HSI + micro-CT + NIR-ONE model achieved a balanced accuracy of approximately 0.442. Multimodal fusion therefore did not outperform NIR-ONE alone in the corrected five-class comparison. Raman was excluded from the five-class models because class 5 was not quantitatively evaluable. These findings further support the use of task-specific diagnostic contrasts.

### 3.9. Task-Specific Diagnostic Decision-Support Framework

Based on the task-specific internal results, a hypothesis-generating exploratory decision-support framework was constructed. It should not be interpreted as a validated sequential classifier, forensic guideline, or legal decision rule. Samples of unknown provenance should first undergo conservative pre-analytical handling, documentation, and rapid non-destructive optical screening using HSI and/or NIR-ONE spectroscopy. When the principal question is whether a sample is compatible with substantially older archaeological material rather than the later forensic C4 interval, high-wavelength NIR-ONE information, particularly reflectance at 1944 nm, provided the strongest rapid optical evidence in the direct exploratory C4-versus-C5 analysis. A strong C5-like pattern may subsequently undergo structural assessment using micro-CT, for which Mean2 showed the strongest performance in both the pooled and direct C4-versus-C5 analyses. HSI-derived TWI remained highly informative for pooled C5-versus-C1–C4 discrimination but was less effective for specifically resolving C4 from C5 and should therefore be regarded as complementary rather than definitive for this boundary.

When the forensic question concerns differentiation between early and later PMI intervals, targeted assessment may be performed using the device-derived HSI StO_2_ and TWI indices, with the NIR spectral slope serving as complementary information. Raman spectroscopy is positioned as a molecular characterisation technique within classes 1–4 because excessive fluorescence prevented reliable quantitative analysis of class-5 spectra. Class 1-versus-class 2 estimates should be reported probabilistically rather than as definitive classifications. Samples with discordant analytical findings, inadequate signal quality, or patterns compatible with the transitional C3 interval should remain indeterminate rather than being forced into an early or later category. Final interpretation should distinguish between potentially medico-legally relevant, indeterminate, and historical/archaeological-compatible analytical patterns and should explicitly report uncertainty and analytical limitations. The resulting framework is an exploratory decision-support proposal rather than an independently validated sequential classifier.

More specifically, a measurement should be considered non-evaluable for the corresponding modality when signal quality is insufficient for reliable analysis, when fluorescence prevents Raman peak extraction, when HSI regions are dominated by glare, shadow, contamination, or damage, when sample preparation is uncertain, or when measured values fall outside the range represented in the present dataset. Discordant results across modalities should likewise trigger an indeterminate decision rather than a forced PMI assignment. In such cases, analytical quality and contextual information should be reassessed and, where appropriate, an orthogonal modality may be used for additional characterisation. Numerical operating points derived in the present dataset should not be used as stand-alone forensic casework thresholds before independent external validation.

The task-specific diagnostic performance, proposed analytical roles and principal limitations of the most informative parameters are summarised in Table 1.

## 4. Discussion

The principal finding of the present study is that the diagnostic value of multimodal imaging and spectroscopy for post-mortem interval assessment is strongly task-dependent. Rather than identifying one universally superior modality or one optimal global five-class model, the present reanalysis demonstrated distinct modality-specific strengths for different forensic questions. Micro-CT Mean2, NIR-ONE reflectance at 1944 nm, and HSI-derived TWI showed strong internal performance for distinguishing archaeological class 5 from the pooled C1–C4 reference group. Importantly, the additional exploratory direct C4-versus-C5 analysis refined this interpretation: micro-CT Mean2 showed complete separation of the available samples (AUC 1.000), and NIR-ONE reflectance at 1944 nm retained excellent performance (AUC 0.963), whereas HSI-derived TWI showed only moderate direct discrimination (AUC 0.742). Thus, the modality ranking depended not only on whether archaeological material was included, but also on the precise forensic boundary being assessed. Conversely, the device-derived HSI StO_2_ and TWI indices were most informative for differentiating early classes 1 + 2 from later classes 4 + 5, while Raman-derived mineral and matrix parameters provided complementary molecular information within classes 1–4. Discrimination between class 1 and class 2 remained moderate, and corrected global five-class modelling did not consistently outperform the strongest single-modality model. Taken together, these findings indicate that PMI assessment should be organised according to the specific forensic decision task rather than as a uniform all-class classification problem.

Post-mortem bone alteration is multidimensional. Structural degradation, mineral-density changes, alterations in collagen and organic matrix, hydration changes, microbial effects, surface heterogeneity and environmental exposure do not occur uniformly and are not captured equally by any single analytical method. Micro-CT provides information on cortical architecture and density-related features. HSI provides spatial surface-level optical information. Mira Raman spectroscopy provides molecular mineral and matrix information, particularly in phosphate-associated spectral regions around 953–960 cm^−1^. NIR-ONE spectroscopy provides rapid, point-based near-infrared information on hydration, organic matrix composition, and mineral-associated properties. The observed task dependence is biologically plausible because different stages of post-mortem alteration affect bone structure, hydration, optical properties and molecular composition to different extents.

Very-old and archaeological bone may exhibit pronounced density-related and structural changes together with distinct high-wavelength NIR and HSI-derived TWI spectral signatures, which were captured particularly well by micro-CT, NIR-ONE, and HSI in the present dataset. By contrast, differences between early and later PMI intervals may be reflected more strongly in the device-derived StO_2_ and water-related optical indices generated by HSI. In the context of post-mortem, air-dried skeletal material, these HSI-derived parameters should not be interpreted as physiological measures of tissue oxygenation, perfusion, haemoglobin content or hydration. Rather, they represent algorithm-derived spectral indices whose variation may reflect PMI-associated changes in surface optical properties, water-related spectral characteristics, tissue composition and post-mortem matrix alteration. Raman spectroscopy interrogates phosphate-, carbonate-, crystallinity- and matrix-associated changes and therefore provides complementary molecular information within the Raman-compatible forensic range.

An important interpretive limitation is that C5 differs from the remaining classes not only in assigned PMI but also in archaeological and taphonomic context. Chronological age therefore cannot be disentangled from burial environment, preservation state, microbial exposure, storage conditions, and post-recovery history in the present cohort. The observed C5-associated structural and spectral signatures must consequently be interpreted as archaeological-compatible, context-dependent patterns rather than as biomarkers of elapsed time alone. Furthermore, the classification system contains a substantial unrepresented interval between C4 (>1–10 years) and C5 (>100 years). The present data therefore cannot establish a continuous temporal transition or a validated chronological threshold between later forensic and archaeological material. Future studies should incorporate structured taphonomic metadata and skeletal remains spanning the currently unrepresented interval between approximately 10 and 100 years.

No single modality can be expected to capture all these processes equally well across the full PMI spectrum. This interpretation is consistent with recent reviews that emphasise that late PMI estimation remains unresolved because multiple intrinsic and environmental factors influence skeletal decomposition and diagenesis, and that no single, universally accepted method currently provides reliable dating across broad forensic and archaeological intervals [1,3]. It is also consistent with histological evidence showing that bone diagenesis involves early collagen alteration and context-dependent microscopic degradation [4].

The novelty of the present study lies in the harmonisation of previously modality-specific datasets at the level of the unique physical bone sample and in their direct comparison across defined forensic decision tasks. Rather than reassessing whether each individual modality contains PMI-associated information, the present analysis integrates structural, molecular, point-spectroscopic and spatial optical information within a common sample-level analytical framework. In particular, the study newly combines interpretable HSI-derived ROI parameters, wavelength-specific NIR-ONE features, predefined micro-CT parameters and molecular Raman markers in task-specific diagnostic comparisons.

A further methodological contribution is the aggregation of repeated measurements, spectra and regions of interest at the physical-sample level before statistical analysis and model development, thereby reducing the risk of information leakage from technical replicates. Importantly, the corrected analyses also demonstrated that multimodal fusion did not inherently improve diagnostic performance. The strongest global five-class multimodal model did not outperform the best single-modality model, whereas individual modalities showed distinct strengths for specific forensic contrasts. Thus, the added value of multimodality in the present study lies not in universal feature fusion, but in the selective use of complementary analytical information according to the forensic question being addressed.

A central finding of the present reanalysis is the added value of HSI-derived ROI features. In earlier HSI work, classification was based on deep-learning analysis of hyperspectral image data. In the present study, the available standardised HSI parameter maps were reprocessed into sample-level ROI features, including NIR perfusion, oxygenation/StO_2_, tissue haemoglobin index, and tissue water index.

The targeted analysis showed, however, that the value of HSI was not limited to multimodal feature fusion. HSI-derived StO_2_ achieved an AUC of 0.940 for classes 1 + 2 versus classes 4 + 5, and TWI achieved an AUC of 0.894. Thus, HSI was the strongest modality for the early-versus-later PMI contrast. This suggests that the spectral characteristics captured by the device-derived HSI StO_2_ index and TWI indices may change earlier or more consistently than gross structural features during the transition from early to later PMI. These changes should be interpreted as alterations in post-mortem optical and compositional properties rather than as changes in physiological tissue oxygenation or perfusion. The exploratory Perfusion/(Perfusion + THI) index showed directional class-related behaviour but was less informative than StO_2_ and TWI and should therefore not be prioritised as a primary diagnostic marker. These findings support a direct task-specific role for HSI in early-versus-later PMI assessment, in addition to its complementary value in multimodal models.

The targeted archaeological analysis provided a more forensically interpretable assessment than the one-vs-rest sensitivity and specificity derived from the previous global five-class model. Micro-CT Mean2 achieved an AUC of 0.984, NIR-ONE reflectance at 1944 nm an AUC of 0.980 and HSI TWI an AUC of 0.961 for class 5 versus classes 1–4. At exploratory operating points requiring approximately 95% specificity, micro-CT Mean2 retained a sensitivity of 1.000, whereas NIR-ONE 1944 nm and HSI TWI each retained a sensitivity of approximately 0.833. These findings address the forensically relevant trade-off between avoiding false archaeological assignments and preserving sensitivity. Nevertheless, class 5 comprised only six unique physical samples. The resulting confidence intervals, sensitivity estimates and operating points are therefore unstable and must not be interpreted as validated forensic thresholds.

The distinction between later forensic C4 and archaeological C5 material deserves particular consideration because it represents a forensically relevant temporal boundary within the present classification scheme. Class 4 comprised skeletal remains with an assigned PMI of 1–10 years, whereas class 5 represented archaeological material older than 100 years. Although the primary archaeological analysis compared C5 with the pooled C1–C4 reference group rather than with C4 alone, C4 constituted the temporally closest forensic class included in that comparator group. The high discriminatory performance observed for C5 therefore indicates that the C5-associated structural and optical pattern remained detectable despite the inclusion of later forensic C4 samples.

From a forensic triage perspective, this finding is particularly relevant for skeletal remains of unknown provenance, for which one of the first analytical questions may be whether the observed pattern is compatible with a potentially medico-legally relevant forensic interval or with a substantially older, archaeological context. The exploratory direct C4-versus-C5 analysis showed complete separation for micro-CT Mean2 in the available samples (AUC 1.000; C4, *n* = 10; C5, *n* = 5) and excellent discrimination for NIR-ONE reflectance at 1944 nm (AUC 0.963; 95% bootstrap CI, 0.833–1.000; C4, *n* = 9; C5, *n* = 6), whereas HSI-derived TWI showed only moderate discrimination (AUC 0.742; 95% bootstrap CI, 0.475–0.983; C4, *n* = 10; C5, *n* = 6). These findings refine the pooled C5-versus-C1–C4 analysis and demonstrate that performance at the specific C4–C5 boundary is modality dependent. Because class sizes were small and the evaluated parameters were selected following the pooled analysis, these findings should be regarded as hypothesis-generating and require independent replication in larger cohorts before any threshold-based forensic application.

The role of Senterra Raman microscopy should be interpreted cautiously. In the targeted analysis, Senterra-derived carbonate/phosphate and Amide III parameters showed potentially useful discrimination within classes 1–4. However, the apparently strong performance of the A577/A958 ratio should be regarded as exploratory because this parameter was identified post hoc and was not a prespecified primary endpoint. Senterra provided complementary high-resolution Raman information but showed lower stand-alone robustness than the primary Mira Raman feature set in the present sample-wise comparison. This finding does not argue against microscopic Raman spectroscopy in general but indicates that improved fluorescence management, harmonised preprocessing, prespecified feature selection and independent replication are required.

A further important finding was the limited applicability of Raman spectroscopy to archaeological class-5 samples. Excessive fluorescence obscured the Raman signal and prevented reliable quantitative peak extraction. Raman was therefore excluded from all quantitative tasks involving class 5. This limitation is analytically relevant because it demonstrates that the absence of usable Raman parameters is itself dependent on sample age, preservation and diagenetic state. Raman spectroscopy should consequently be positioned as a molecular characterisation technique within classes 1–4 rather than as a universal modality across the entire PMI range. Within this restricted range, crystallinity and carbonate/phosphate parameters provided complementary information for differentiating classes 1 + 2 from class 4, but class 1 versus class 2 remained only moderately separable.

NIR-ONE spectroscopy showed distinct task-specific strengths. Reflectance at 1944 nm was highly informative for class-5 discrimination against the pooled C1–C4 reference group and retained excellent performance in the exploratory direct C4-versus-C5 comparison (AUC 0.963). The spectral slope between 1550 and 1950 nm, in contrast, contributed primarily to early-versus-later differentiation. The wavelength-resolved analysis further indicated that the 1944-nm finding was located within a broader discriminatory high-wavelength region rather than representing an isolated single-wavelength effect.

**Figure 7:**
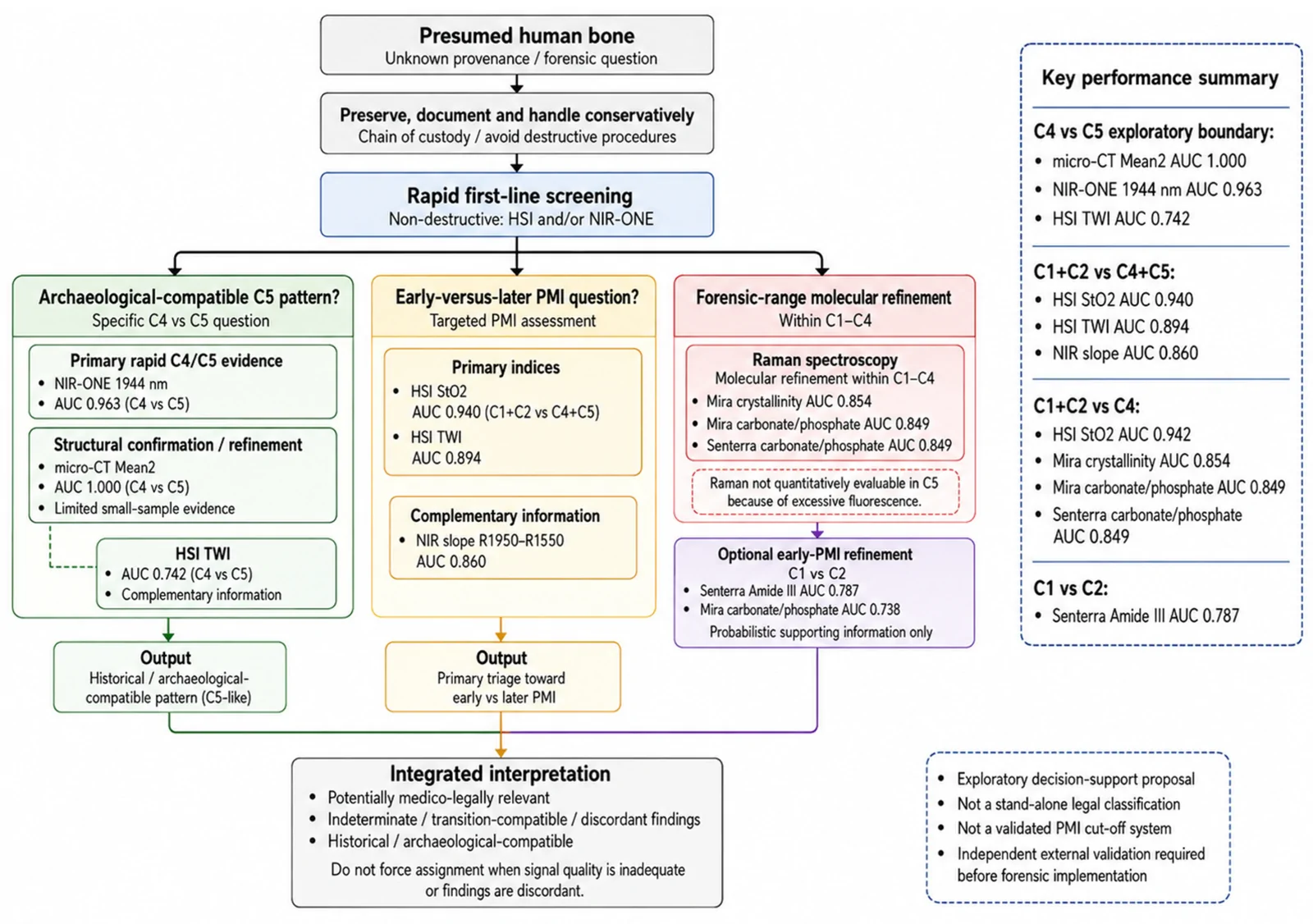
Proposed exploratory task-specific diagnostic decision-support framework for PMI assessment of human skeletal remains. Presumed human bone of unknown provenance is initially preserved, documented, and screened non-destructively using HSI and/or NIR-ONE. For the exploratory C4-versus-C5 boundary, NIR-ONE reflectance at 1944 nm provides rapid optical evidence, micro-CT Mean2 provides structural confirmation, and HSI-derived TWI contributes complementary information. Early-versus-later PMI assessment is primarily supported by HSI-derived StO_2_ and TWI, with the NIR spectral slope as complementary information. Raman spectroscopy provides molecular refinement within C1–C4, while C1-versus-C2 assessment is considered probabilistic only. Discordant or low-quality findings should remain indeterminate. The framework is a hypothesis-generating exploratory decision-support proposal and is neither a validated PMI cut-off system, an independently validated sequential classifier, nor a stand-alone legal classification. Discordant, out-of-range, or technically inadequate findings should remain indeterminate, and independent external validation is required before forensic implementation.

From a practical forensic perspective, the decision-support pathway shown in Figure 7 supports selective escalation rather than a fixed requirement to apply every modality to every sample. HSI and NIR-ONE are suitable for rapid, non-destructive first-line screening, but the subsequent analytical step should depend on the forensic question. A strong C5-like optical or NIR pattern may warrant structural confirmation using micro-CT. In contrast, a non-C5 or uncertain sample requiring differentiation within the forensic range may benefit from Raman-based molecular characterisation. Micro-CT is therefore not invariably the final step, and Raman is not universally the second step. The optimal sequence is task-dependent.

For bone samples of unknown provenance, conservative pre-analytical handling remains essential. The sample should initially be treated as potentially medico-legally relevant, destructive procedures should be avoided, contextual information should be documented and chain-of-custody requirements should be maintained. Within this conservative pathway, the analytical results should support one of three interpretive categories: potentially medico-legally relevant, indeterminate, or compatible with historical/archaeological origin. The wording “compatible with” is important because the analytical framework cannot independently establish archaeological provenance or legal status. Discordant results, insufficient signal quality, fluorescence interference, values outside the observed training range or uncertain sample preparation should result in an indeterminate classification rather than a forced PMI assignment.

Several limitations must be considered. First, the study was retrospective and internally evaluated. Although sample-wise harmonisation was used to reduce the risk of data leakage, external validation in independent cohorts is still required. This is particularly relevant because transparent reporting and independent validation are central requirements for prediction-model and AI-assisted diagnostic studies, as emphasised by TRIPOD + AI and imaging-AI reporting guidance [13,14]. Second, the dataset was imbalanced, with relatively few samples in the later PMI classes, especially class 5. Class 5 comprised only six unique physical samples. Consequently, the very high AUC estimates and exploratory operating points for archaeological discrimination may be optimistic and are associated with considerable uncertainty. Accordingly, AUC values approaching 1.0 in the present dataset should not be interpreted as evidence of near-perfect performance in independent forensic material. They represent internally derived hypothesis-generating estimates whose stability must be established in substantially larger and independent C5 cohorts. This limits the stability of class-wise sensitivity estimates and reduces the precision with which performance for archaeological material can be estimated.

More generally, the relatively small cohort, class imbalance, and predictor dimensionality create a risk of model instability and overfitting that cannot be eliminated by internal cross-validation or bootstrap resampling alone. Although the effective dimensionality of the multivariate models was deliberately constrained through predefined low-dimensional feature sets for micro-CT, HSI, and Raman spectroscopy and through training-fold PCA for NIR-ONE, these procedures primarily reduce rather than eliminate the risk of optimistic performance estimation. The primary HSI model contained four sample-level optical indices, the primary micro-CT and Raman models contained six predefined parameters each, and the 201-variable NIR-ONE spectra were reduced to 10 principal components before classification. Nevertheless, the number of independent physical samples remained limited relative to the complexity and heterogeneity of the analytical data, particularly for the smallest PMI classes. Consequently, the reported cross-validated classification metrics and parameter-wise AUC estimates may remain optimistic until replicated in larger independent cohorts. Internal resampling should therefore be regarded as an assessment of model stability within the present dataset rather than as evidence of external generalisability.

In addition, although the exploratory direct C4-versus-C5 analysis provided important information on this specific boundary, it was based on very small class sizes, particularly for C5, and the evaluated parameters were selected following the preceding pooled C5-versus-C1–C4 analysis. The corresponding AUC estimates may therefore be optimistic and should be interpreted as hypothesis-generating rather than as independently validated performance estimates. The complete separation observed for micro-CT Mean2 in the available samples is particularly susceptible to instability in a small dataset and should not be interpreted as evidence of perfect diagnostic performance in independent material. Third, the modalities differed in acquisition structure, preprocessing, dimensionality, and feature availability. Although harmonisation was performed at the physical-sample level, the individual modalities were not acquired prospectively under one uniform protocol. Differences in acquisition date, sample preparation, device characteristics, preprocessing and missingness may therefore have influenced apparent cross-method performance. Furthermore, modality-specific analyses were based on partly different analysis-ready subsets. Consequently, differences in apparent performance between modalities may partly reflect differences in sample availability and subset composition. The cross-method results should therefore be interpreted as task-specific comparative evidence rather than as a definitive matched-sample ranking of analytical technologies. A further limitation concerns incomplete donor-level baseline metadata. Because the present study retrospectively harmonised several modality-specific legacy datasets, donor age, sex, detailed skeletal disease or pathology, and a harmonised numerical observation-angle variable were not consistently available for all 107 unique physical samples. These variables were therefore neither imputed nor inferred, and their potential influence on the observed structural, molecular, or optical differences could not be assessed systematically. Anatomical sampling was more consistent, as the harmonised cohort comprised femoral diaphyseal cortical bone samples prepared as transverse sections, and HSI acquisition was performed under standardised conditions at a fixed camera-to-sample distance of 50 cm. Nevertheless, residual confounding by donor demographic characteristics, skeletal pathology, or acquisition geometry cannot be excluded. Future prospective studies should therefore collect these covariates systematically and evaluate their potential effects on PMI-associated analytical signatures. Environmental and taphonomic metadata were also not available in sufficient detail to model their influence systematically. Burial conditions, temperature, humidity, soil composition, microbial exposure, storage conditions and post-recovery handling may produce spectral or structural changes that overlap with PMI-associated effects [3,5]. Furthermore, PMI class and sample context were not fully separable at the oldest end of the dataset, because class-5 material originated from archaeological contexts. Consequently, differences attributed to the C5 interval may reflect a combination of chronological age, burial history, preservation conditions, environmental exposure, and other taphonomic factors. The present analytical signatures should therefore be interpreted as archaeological-compatible patterns rather than as age-specific biomarkers or proof of archaeological provenance. The classification scheme also contains a substantial temporal gap between C4 (>1–10 years) and C5 (>100 years). Therefore, the present results do not establish how the identified structural or spectral patterns behave in remains with PMIs between approximately 10 and 100 years, and the C4-versus-C5 analysis should not be interpreted as defining a continuous temporal threshold between forensic and archaeological material.

An additional analytical limitation is that quantitative Raman analysis was not possible in class 5 because of excessive fluorescence, preventing direct cross-modality comparison across the full PMI range. Sixth, wavelength-specific NIR features and exploratory Raman ratios were identified within the present dataset and require independent replication. Seventh, the operating points requiring approximately 95% specificity were internally derived and were not prespecified or externally calibrated. Eighth, discrimination between class 1 and class 2 remained moderate and should not be interpreted as a reliable binary forensic test. Ninth, model calibration, inter-device reproducibility, inter-operator reproducibility and prospective out-of-distribution performance were not evaluated.

Future studies should use prospective multicentre cohorts, balanced PMI distributions, harmonised acquisition protocols, donor- or case-wise validation, prespecified parameters and thresholds, structured taphonomic metadata, representation of intermediate late-PMI intervals, and independent external test sets. In particular, the exploratory C4-versus-C5 findings should be replicated in larger cohorts before the proposed modality-specific decision pathway is translated into forensic casework. The framework should therefore be regarded as an internally evaluated exploratory decision-support proposal rather than a forensic guideline or legal classification standard.

In summary, the present study advances previous modality-specific work by demonstrating that diagnostic performance depends on the forensic question being addressed. Micro-CT Mean2 and high-wavelength NIR-ONE reflectance were particularly informative for archaeological class-5 discrimination and retained excellent performance in the exploratory direct C4-versus-C5 analysis. HSI-derived StO_2_ and TWI indices were strongest for early-versus-later PMI assessment, whereas HSI TWI was less informative for specifically resolving the C4–C5 boundary. Raman-derived molecular parameters provided complementary information within classes 1–4. Class 1 versus class 2 remained difficult, and corrected global five-class fusion did not consistently outperform the strongest single-modality approach. The resulting framework therefore recommends task-specific modality selection, explicit uncertainty reporting, and selective analytical escalation rather than universal application of all methods.

## 5. Conclusions

The diagnostic value of multimodal imaging and spectroscopy for PMI assessment was strongly task-dependent in the present internally evaluated cohort. Micro-CT Mean2 and high-wavelength NIR-ONE reflectance were particularly informative for discrimination of the available archaeological-compatible C5 samples from the pooled C1–C4 reference group. In the additional exploratory C4-versus-C5 analysis, micro-CT Mean2 showed complete observed separation in the available samples and NIR-ONE reflectance at 1944 nm retained strong discrimination, whereas HSI-derived TWI showed only moderate performance for this specific boundary. Given the very small C5 sample size and the confounding of chronological age with archaeological and taphonomic context, these findings should be regarded as hypothesis-generating and not as validated chronological PMI performance estimates.

For the deliberately separated early-versus-later contrast, the device-derived HSI StO_2_ and TWI indices were the most informative parameters, while Raman-derived mineral and matrix features provided complementary molecular information within C1–C4. Exclusion of the transitional C3 interval may increase apparent separation and therefore limits extrapolation of these estimates to a continuous or unselected PMI population. Class 1-versus-class 2 discrimination remained moderate, and corrected global five-class multimodal fusion did not consistently outperform the strongest single-modality approach.

Taken together, the findings support further investigation of task-specific modality selection and selective analytical escalation rather than uniform application of all modalities. The proposed framework is a hypothesis-generating exploratory decision-support concept derived from internal analyses and is not a validated sequential classifier, forensic guideline, or legal decision rule. Prospective multicentre studies with larger and more balanced cohorts, structured taphonomic metadata, representation of intermediate late-PMI intervals, harmonised acquisition protocols, and independent external test sets are required before numerical thresholds or the proposed analytical pathway can be considered for forensic casework.

## Figures and Tables

**Table 1:** Task-specific diagnostic performance and proposed interpretation of multimodal PMI assessment.

Diagnostic Task	Comparison	Most Informative Modality or Parameter	Internal Performance	Proposed Diagnostic Role	Important Limitation
Exploratory discrimination of archaeological-compatible C5 material	C5 vs. C1–C4	micro-CT Mean2	AUC 0.984; sensitivity 1.000 at specificity 0.980	Structural confirmation of a strong C5-like pattern	C5 comprised only 5 samples with valid Mean2 data; threshold not externally validated
	C5 vs. C1–C4	NIR-ONE reflectance at 1944 nm	AUC 0.980; sensitivity 0.833 at specificity 0.989	Rapid, non-destructive screening for a C5-like spectral pattern	Wavelength-specific result requires independent external and inter-device validation
	C5 vs. C1–C4	HSI tissue water index (TWI)	AUC 0.961; sensitivity 0.833 at specificity 0.960	Spatial optical screening for archaeological-compatible changes	Surface condition and taphonomic factors may influence the device-derived optical index
Exploratory later-forensic versus archaeological-compatible C5 boundary	C4 vs. C5	micro-CT Mean2	AUC 1.000; complete separation in available samples (C4, *n* = 10; C5, *n* = 5)	Structural confirmation/refinement of the specific C4–C5 boundary	Very small sample size; complete separation may be unstable and must not be interpreted as perfect diagnostic performance
	C4 vs. C5	NIR-ONE reflectance at 1944 nm	AUC 0.963 (95% CI 0.833–1.000); sensitivity 0.833 at specificity 1.000	Rapid optical evidence for differentiation of later forensic from archaeological-compatible material	Small C5 sample and post-analysis parameter selection; requires independent replication
	C4 vs. C5	HSI TWI	AUC 0.742 (95% CI 0.475–0.983)	Complementary optical information	Only moderate direct C4–C5 discrimination despite strong pooled C5-versus-C1–C4 performance
Early-versus-later PMI discrimination	C1 + C2 vs. C4 + C5; C3 excluded	HSI oxygenation/StO_2_ index	AUC 0.940; sensitivity 0.625 at specificity 0.975	Primary optical parameter for targeted early-versus-later assessment	Device-derived index; not a physiological oxygen-saturation measurement; operating point requires external validation
	C1 + C2 vs. C4 + C5; C3 excluded	HSI TWI	AUC 0.894; sensitivity 0.813 at specificity 0.950	Complementary HSI parameter for early-versus-later assessment	Device-derived optical index; not a stand-alone PMI marker
	C1 + C2 vs. C4 + C5; C3 excluded	NIR-ONE spectral slope R1950–R1550	AUC 0.860	Complementary point-spectroscopic information	May depend on acquisition geometry, device characteristics and sample preparation
Forensic-range discrimination	C1 + C2 vs. C4	HSI oxygenation/StO_2_ index	AUC 0.942	Strong optical evidence for differentiation within the forensic range	Device-derived optical index; independent validation required
	C1 + C2 vs. C4	NIR-ONE spectral slope R1950–R1550	AUC 0.865	Complementary non-destructive spectral information	Exploratory wavelength-derived parameter requiring independent replication
Forensic-range molecular refinement	C1 + C2 vs. C4	Mira Raman crystallinity	AUC 0.854	Molecular assessment of mineral organisation within C1–C4	Raman was not quantitatively evaluable in C5
	C1 + C2 vs. C4	Mira Raman carbonate/phosphate ratio	AUC 0.849	Complementary mineral-composition information	Requires harmonised preprocessing and independent replication
	C1 + C2 vs. C4	Senterra Raman carbonate/phosphate ratio	AUC 0.849	Exploratory high-resolution molecular characterisation	Senterra results were less robust as a complete stand-alone feature set
Early-PMI refinement	C1 vs. C2	Senterra Amide III	AUC 0.787	Probabilistic supporting molecular information	Moderate performance; does not support definitive binary classification
	C1 vs. C2	Mira Raman carbonate/phosphate ratio	AUC 0.738	Complementary molecular evidence	Considerable overlap between the two early PMI classes
Global five-class benchmark	C1–C5	NIR-ONE SNV/PCA model	Balanced accuracy ≈ 0.483	Secondary benchmark across the complete PMI range	Moderate performance; not suitable as a definitive five-class forensic classifier
	C1–C5	HSI + micro-CT + NIR-ONE	Balanced accuracy ≈ 0.442	Exploratory multimodal benchmark	Multimodal fusion did not outperform NIR-ONE alone

Abbreviations: AUC, area under the receiver operating characteristic curve; C1–C5, post-mortem interval classes 1–5; CI, confidence interval; HSI, hyperspectral imaging; NIR, near-infrared spectroscopy; PCA, principal component analysis; SNV, standard normal variate; StO_2_, device-derived oxygenation index; TWI, tissue water index. Note: All parameter-wise performance estimates represent internal evaluation of the present dataset. Operating points were selected exploratorily at approximately 95% specificity and should not be interpreted as externally validated forensic cut-offs. The direct C4-versus-C5 analysis was exploratory because of the small C5 sample size and because the evaluated parameters were selected following the pooled C5-versus-C1–C4 analysis. Independent external validation is required before forensic implementation. C5-related analytical patterns should be interpreted as archaeological-compatible rather than age-specific because chronological PMI and archaeological/taphonomic context are not separable in the present cohort.

## Data Availability

The data presented in this study are available upon request from the corresponding author.

## Abbreviations

The following abbreviations are used in this manuscript:

BV/TV	Bone volume fraction
CT	Computed tomography
HSI	Hyperspectral imaging
micro-CT	Micro-computed tomography
NIR	Near-infrared
NIR-ONE	Near-infrared spectroscopy platform
PCA	Principal component analysis
PMI	Post-mortem interval
RGB	Red-green-blue
ROI	Region of interest
SD	Standard deviation
StO_2_	Device-derived oxygenation index
SVM	Support vector machine
THI	Tissue haemoglobin index
TWI	Tissue water index
